# Behavior of Electrothermal Actuator Analyzed by Polynomial Point Interpolation Collocation Method

**DOI:** 10.3390/mi16121415

**Published:** 2025-12-16

**Authors:** Yujuan Tang, Aidong Qi, Yuanhu Gu, Yinfa Zhu, Haojie Li, Dao Gu, Hao Chen

**Affiliations:** 1 School of Intelligent Science and Control Engineering, Jinling Institute of Technology, Nanjing 211169, China; yjtang@jit.edu.cn; 2 Zhejiang Jiali (Lishui) Industrial Co., Ltd., Lishui 323000, China; lishuijiali@vip.163.com; 3 Artificial Intelligence School, Lishui University, Lishui 323000, China; 18921884431@189.com; 4 State Key Laboratory of Fluid Power and Mechatronic Systems, Zhejiang University, Hangzhou 310027, China; 5 Lishui Key Laboratory of High Power Density Intelligent Drive System, Lishui 323000, China; 6 Engineering School, Lishui University, Lishui 323000, China; lszhuyinfa@163.com; 7 State Key Laboratory of Precision Manufacturing for Extreme Service Performance, Central South University, Changsha 410000, China; 8 Lishui Zhe Li Digital Resources Management Co., Ltd., Lishui 323000, China; miaojia527@163.com; 9 Faculty of Engineering and Information Technology, The University of Technology Sydney, Ultimo, NSW 2007, Australia; gudao901@gmail.com

**Keywords:** polynomial point interpolation collocation method, thermo-mechanical response, microactuator meshfree

## Abstract

This paper presents a novel implementation of the Polynomial Point Interpolation Collocation Method (PPCM) for analyzing the coupled electrothermal and thermomechanical behavior of V-shaped microactuators. Within the PPCM framework, the governing equations for heat transfer and structural mechanics are discretized over the computational domain. The resulting discrete electrothermal system is solved in a fully coupled manner via an incremental load method to determine the temperature field. Subsequently, the displacement field is computed by solving the discrete mechanical equation, which incorporates terms from the natural boundary conditions. The MQ radial basis function behaves well in convergence when its parameters *p_a_* and *p_q_* are 1 and 1.8. Under a 6 V voltage, the difference between the PPCM and FEM temperature values is less than 1 °C. Meanwhile, the discrepancy between the PPCM and experimental temperature values is approximately 20 °C, corresponding to an approximate error of 10%. Furthermore, the displacement error between the PPCM and FEM is as low as approximately 2 μm under an applied voltage of 12 V. These results validate the PPCM for predicting the driving characteristics of V-shaped microactuators.

## 1. Introduction

Microactuators serve as fundamental components in micro- and nano-electromechanical systems (MEMS and NEMS), enabling diverse applications such as micro-grippers [1], high-precision positioning systems [2,3], and bistable or multistable micro-switches [4,5]. These devices operate by converting electrical signals into mechanical motion through multi-physical interactions. Depending on their operational principles, microactuators are commonly classified into piezoelectric [6], electrostatic [7], electrothermal [8], and electromagnetic [9] types. In particular, electrothermal actuators have garnered significant interest owing to their ability to generate large displacements and high driving forces, along with their compatibility with standard microfabrication processes.

The past few decades have witnessed the fast development of advanced computational techniques for small-scale structural analysis, which continues to attract significant and growing interest within the research community. Ouakad et al. [10]. Predicted the steady-state behavior of an electrostatically actuated and initially curved actuator arrangement with the meshless Galerkin decomposition method. Jankowski et al. [11] considered the vibration of a piezoelectrically actuated smart nanoactuator via nonlocal strain gradient theory on the basis of the meshless differential quadrature method. The work of Chen et al. [12] featured the implementation of the local radial point interpolation method (LRPIM) for electrothermal structure dynamics, where temperature data served to validate the results. Xia et al. [13] completed an electroelastic analysis of piezoelectric structures based on the meshfree generalized finite difference method (GFDM). Nourmohammadi et al. [14] numerically solved the shear deformation problem of a functionally graded piezoelectric plate using the radial point interpolation technique. In addition, the emerging field of data-driven numerical computation integrates data science with traditional computational physics. Its feature is the fusion of physical principles with data to create methods that are less reliant on explicit, closed-form equations. This is often achieved through machine learning, which can reveal hidden patterns and complex nonlinear relationships, leading to a transformative shift in how we simulate and understand complex systems. Machine learning techniques have demonstrated remarkable potential in diverse interdisciplinary applications, including biomedical engineering and soft robotics. For instance, machine learning-enhanced soft robotic systems inspired by biological functions have been developed for medical diagnostics and rehabilitation [15]. While these applications differ in scale and domain from microactuator analysis, they exemplify the transformative potential of data-driven approaches in solving complex, nonlinear engineering problems. This cross-fertilization of ideas motivates the exploration of novel computational frameworks, such as PPCM, that can bridge different physical scales and application domains. Lao et al. [16] employed a Physics-Informed Neural Networks (PINNs) enhanced Deep Reinforcement Learning (DRL) framework for high-precision vibration control of piezoelectric cantilever beams. In order to predict the minimum gap dynamics of an electrostatically actuated microbeam, Barbulescu et al. [17] proposed a data-driven method based on recurrent neural networks.

The classification of meshless methods, according to their solution formulation, falls into two primary types: the strong form and the weak form. The implementation of meshless methods founded on weak formulations [18,19], such as the Galerkin-based approaches or LRPIM, is usually challenging. This is primarily due to the requirement for cumbersome numerical integration over a background mesh and the intricate procedures needed to impose essential boundary conditions, a consequence of their non-interpolating shape functions. Compared with the weak form, the strong formulation, despite its advantages of lower computational expense and a more direct implementation, comes with the drawback of sensitivity to node irregularities and the need for calculation stabilization. This issue is particularly pronounced in the Generalized Finite Difference Method (GFDM) [20,21], where the coefficient matrix from the least-squares fit is highly prone to ill-conditioning. Meanwhile, the PINN, another strong form method, relies on large-scale iterative training and is better adapted for solving inverse problems that incorporate known observational data [22,23]. In addition, the Polynomial Point Interpolation Collocation Method (PPCM) is also a strong-form meshless technique that constructs polynomials to exactly pass through all nodes within local support domains. Given the relatively regular geometry of the electrothermal microactuator, the adoption of PPCM not only ensures numerical stability during computation but also leads to high computational efficiency.

Considering the cumbersome implementation and high computational costs associated with the weak form, as well as addressing limitations such as low accuracy in the Generalized Finite Difference Method (GFDM) and the time-consuming training requirements of Physics-Informed Neural Networks (PINNs), this research focuses on the electrothermal–thermomechanical analysis of V-shaped actuators by employing PPCM to construct an efficient and robust convergent numerical scheme. The paper is structured as follows: Section 2 outlines the fundamental principles of the PPCM and presents a flowchart for the actuator analysis. Section 3 derives the discretized equations by integrating the PPCM with the incremental load method, based on a pre-established electrothermal coupling model. In Section 4, actuator deflection is estimated using the PPCM within a thermo-elastic framework. Section 5 compares the thermal and mechanical results obtained from the PPCM, finite element method (FEM), and experimental measurements and assesses the accuracy and convergence of the PPCM. Finally, Section 6 provides concluding remarks and suggests future research directions.

## 2. Polynomial Point Interpolation Collocation Method

To facilitate comprehension, the shape function constructed by the radial basis polynomial is first derived here. A 2D problem domain **Ω** is discretized into *N* points. Provided that *N^l^* discrete points are identified in the local field of the point **x***_I_*, (*x_I_*, *y_I_*), the field variable vector **u** can be expressed as

(1) u=Ra+Qb

where position vector **x***_I_* contains the *x* and *y*, **a** and **b** are the vectors of undetermined coefficients,

(2) $$u_{s}={\left[u_{1},u_{2},\ldots ,u_{N^{l}}\right]}^{T}$$

and

(3) $$R=\left[\begin{array}{cccc} R_{1}(x_{1}) & R_{2}(x_{1}) & \ldots & R_{N^{l}}(x_{1})\\ R_{1}(x_{2}) & R_{2}(x_{2}) & \ldots & R_{N^{l}}(x_{2})\\ \ldots & \ldots & \ldots & \ldots \\ R_{1}(x_{N^{l}}) & R_{2}(x_{N^{l}}) & \ldots & R_{N^{l}}(x_{N^{l}}) \end{array}\right]$$

where the MQ radial basis function (RBF) is

(4) $$R_{i}(x_{j})=\left\{\begin{array}{cc} (1+{\left(p_{a}*r_{ij}/d\right)}^{p_{q}}, & r_{ij}\leq d\\ 0, & r_{ij}>d \end{array}\right.$$

where the distance *r_ij_* = |**x***_i_* − **x***_j_*|_2_ and *d* is the radius of the support domain. To enhance the smoothness of the shape function, the constant and linear terms are added so that

(5) $$Q={\left[\begin{array}{cccc} 1 & 1 & \ldots & 1\\ x_{1} & x_{2} & \ldots & x_{N^{l}}\\ y_{1} & y_{2} & \ldots & y_{N^{l}}\\ x_{1}y_{1} & x_{2}y_{2} & \ldots & x_{N^{l}}y_{N^{l}} \end{array}\right]}^{T}$$

Further, the equation can be rewritten as

(6) $$\tilde{u_{s}}=\left[\begin{array}{c} u_{s}\\ 0 \end{array}\right]=\left[\begin{array}{cc} R & Q\\ Q^{T} & 0 \end{array}\right]\left[\begin{array}{c} a\\ b \end{array}\right]=Ga_{0}$$

For any point **x***_I_* (*x_I_*, *y_I_*), at the current local domain, its field variable *u_I_* can be expressed as

(7) $$u_{I}=\Phi_{I}\tilde{u_{s}}$$

where vector $\Phi_{I}$ is with 1 × (*N^l^* + 4).

(8) $$\Phi_{I}=[\begin{array}{lllllll} R_{1}(x_{I}) & R_{2}(x_{I}) & \cdots & R_{N^{l}}(x_{I}) & 1 & \cdots & x_{I}y_{I}] \end{array}G^{-1}$$

For the convenience of calculation, the last four elements are removed from the vector $\Phi_{I}$ to construct the shape function vector **N***_I,_* in which the dimension is 1 × *N^l^*. Hence, a more unified compact form of Equation (7) can be expressed as

(9) $$u_{I}=N_{I}u_{s}$$

For a 2D boundary value problem, the derivative terms—both within the governing PDE and the accompanying boundary conditions—can be supplanted by differentiating the corresponding linear Equation (9).

## 3. Electrothermal Analysis

**Figure 1:**
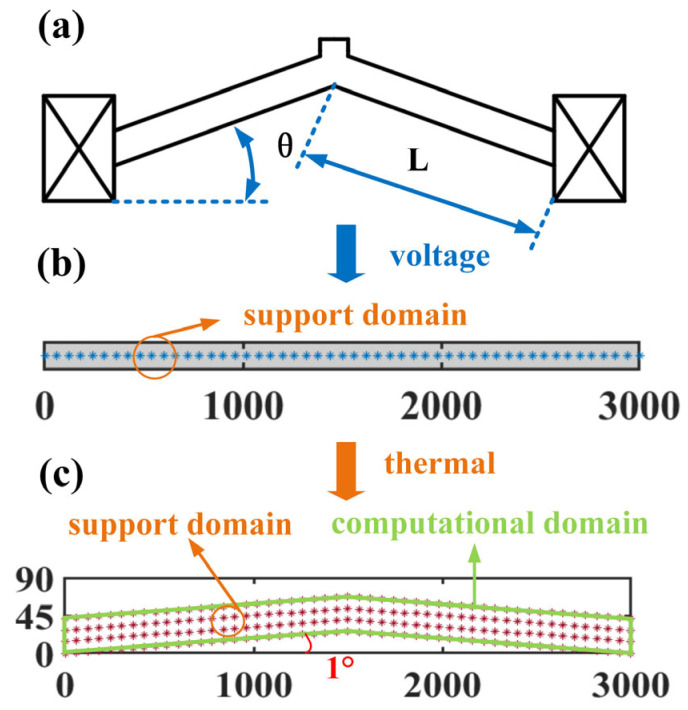
(**a**) Geometric schematic of the V-shaped actuator. (**b**) Discrete node distribution in the electrothermal analysis. (**c**) Discrete node distribution in the thermomechanical analysis.

The geometric model of the V-shaped actuator is shown in Figure 1a. Given the characteristics of the temperature distribution on the actuator, the computational model of the electrothermal analysis can be simplified as a 1D model. Thus, the design of the discrete domain is shown in Figure 1b, where its support domain will be a segment, just related to the x-coordinate. According to the temperature distribution from the 1D model, the discrete node distribution in the thermomechanical analysis is presented in Figure 1c.

According to our previous studies, the coupling equation between the electrical and thermal fields can be derived as

(10) $$k(T)\frac{\partial^{2}T}{\partial x^{2}}-(k_{v}\frac{w+2h}{wh}+\frac{S}{h})T+\frac{U_{0}^{2}\rho (T)}{(\int_{0}^{2l}\rho (T)\;dx{)}^{2}}\;=0$$

where thermal conductivity of polysilicon *k* = 210658(*T* − 30)^−1.2747^ W⸱m^−1^⸱°C^−1^, width *w* and thickness *h* of the actuator are 42 and 100 μm, electrical resistivity *ρ* = 0.0002(1 + 0.003(*T* − 30)) Ω⸱m, the parameters of the heat dissipate rate *k_v_* and *S* are 70 and 0.1(*T* − 30) W⸱m^−2^, and *U*_0_ is the voltage. At both ends of the actuator, the temperatures are forced to be the room temperature. According to the PPCM above, the matrix **Q** in Equation (5) is rewritten as

(11) $$Q={\left[\begin{array}{cccc} 1 & 1 & \ldots & 1\\ x_{1} & x_{2} & \ldots & x_{N^{l}} \end{array}\right]}^{T}$$

Then the vector $\Phi_{I}$ in Equation (7) is with 1 × (*N^l^* + 2). Hence, the last two columns should be removed from the vector $\Phi_{I}$ to construct the shape function vector **N***_I_*. For Equation (8), its discretization form at the interior point *x_I_* should satisfy

(12) $$K^{I}T^{I}=p^{I}$$

where

(13) $$K^{I}=k(T_{I})N_{I,xx}-(k_{v}\frac{w+2h}{wh}+\frac{S}{h})N_{I}$$

and

(14) $$p^{I}=-U_{0}^{2}\rho (T_{I})/(\frac{2l}{N}\sum_{j=1}^{N-1}\rho (T(x_{j})))$$

where $T_{I}$ denotes the temperature at node *x_I_*. **N***_I_*_,*x*_ and **N***_I_*_,*xx*_ denote the first- and second-order derivatives at the *x_I_* versus *x*, respectively. They can be separately obtained from $\Phi_{I,x}$ and $\Phi_{I,xx}$. Here, $\Phi_{I,x}$ and $\Phi_{I,xx}$ can be expressed as

(15) $$\Phi_{I,x}=\left[\begin{array}{llllll} \frac{dR_{1}(x_{I})}{dx} & \frac{dR_{2}(x_{I})}{dx} & \ldots & \frac{dR_{N^{l}}(x_{I})}{dx} & 0 & 1 \end{array}\right]G^{-1}$$

(16) $$\Phi_{I,xx}=\left[\begin{array}{llllll} \frac{d^{2}R_{1}(x_{I})}{dx^{2}} & \frac{d^{2}R_{2}(x_{I})}{dx^{2}} & \ldots & \frac{d^{2}R_{N^{l}}(x_{I})}{dx^{2}} & 0 & 0 \end{array}\right]G^{-1}$$

Thus, this linear vector Equation (12) will be assembled into the total matrix Equation (17)

**Figure 2:**
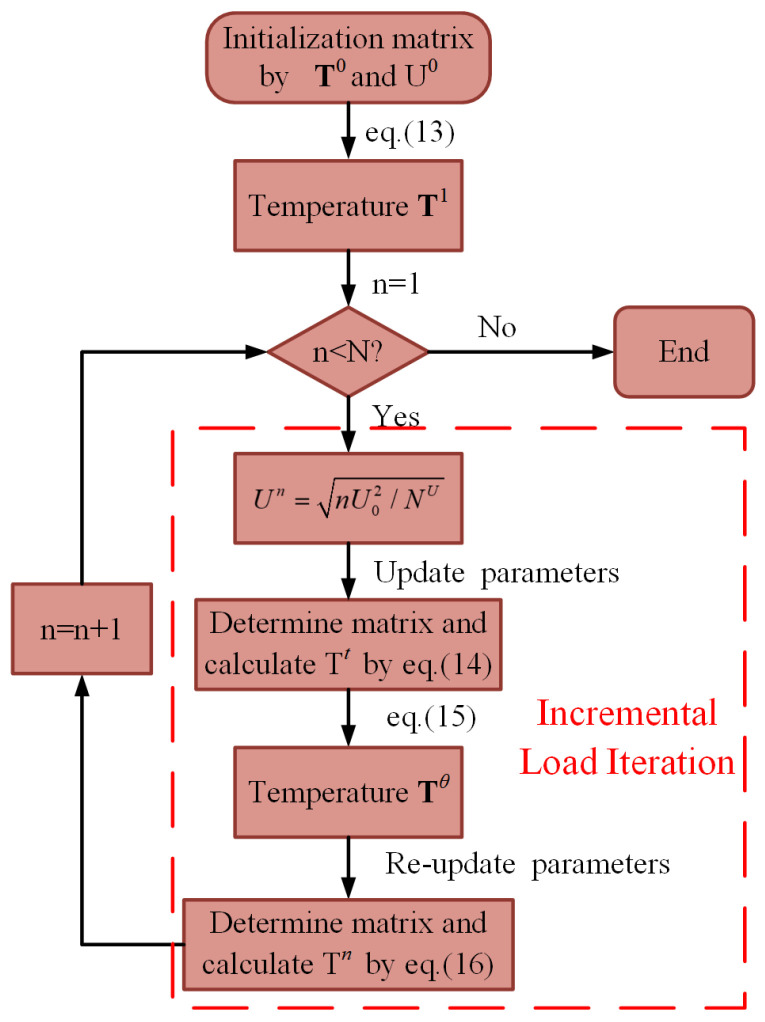
Iteration computation on the electrothermal analysis by the PPCM.

(17) KT=P

where the matrix **K** has dimension of *N^l^* × *N^l^*, vectors, **T** and **P**, are with *N^l^* × 1. For any element $K_{1,j}^{I}$ in the $K^{I}$, the global index assigned to the point *x_I_* is denoted by *I*, while the local index assigned to the point *x_j_* is denoted by *j*. The global index *J* corresponding to the local index *j* is obtained through a mapping. Hence, the element $K_{1,j}^{I}$ is to be added to the global stiffness matrix K at *I*th row and *J*th column. Similarly, the element $T_{j}^{I}$ in the $T^{I}$ is to be added in the *J*th row of the vector **T**. The *p_I_* is assembled into the *I*th row of vector **P**. Once assembly is complete, the essential boundary condition is imposed at the two boundary points. Because of the highly nonlinear parameters *ρ* and *k* varying with the temperature, the incremental load iteration needs to be applied to solve Equation (16). In the iteration computation, an equal increment of the input power, U = $\sqrt{U_{0}^{2}/N^{U}}$, is constantly implemented for each iteration step. In it, *N^U^* is the number of iterations. The procedure of the computational iteration is shown in Figure 2, and the detailed iteration procedure is as follows:

(i) Set the initial values for the nonlinear material parameters and then calculate the matrix K, inverse matrix **K**^−1,^ and vector **P**^1^. From these, the temperature **T**^1^ at the first iteration is computed by Equation (17).
(ii) Update the applied voltage *U^n^* and material parameters based on the temperature of the previous iterative step, where *U^n^* = $\sqrt{nU_{0}^{2}/N^{U}}$. After that, the matrixes K, $K^{-1}$, and vector $P^{n}$ are obtained based on the temperature from the previous iteration T^n−1^. To avoid the accumulation of computational error, the transition temperature **T***^t^* is determined from Equation (18).
  (18) $$T^{t}=K^{-1}(P^{n}-K*T^{n-1})+T^{n-1}$$
(iii) Further refine the transition temperature by
  (19) $$T^{\theta }=(1-\theta )T^{n}+\theta T^{t},\theta =0.9$$
(iv) Renewed the matrix $K^{-1}$ and vector $P^{n}$ using **T***^θ^*. Finally, the temperature **T***^n^* at the nth iteration is computed via Equation (20), from which the next iteration proceeds.
  (20) $$T^{n}=K^{-1}(P^{n}-P^{n-1})+T^{n-1}$$
(v) Termination condition: If the current iteration count reaches or exceeds the maximum allowed, terminate the iteration. Otherwise, proceed to step (ii).

## 4. Thermomechanical Analysis

Combining the isotropic thermal expansion coefficient with the temperature distribution obtained from the above analysis, the thermal strain profile can be further calculated based on the discrete domain in Figure 1c. Hence, the steady-state thermoelastic equation is further presented as

(21) $$\left\{\begin{array}{c} \frac{E}{1-\mu^{2}}(\frac{\partial^{2}u}{\partial x^{2}}+\frac{1-\mu }{2}\frac{\partial^{2}u}{\partial y^{2}}+\frac{1+\mu }{2}\frac{\partial^{2}v}{\partial x\partial y}-\frac{\partial \varepsilon_{x}^{t}}{\partial x}-\mu \frac{\partial \varepsilon_{y}^{t}}{\partial x})=0\\ \frac{E}{1-\mu^{2}}(\frac{\partial^{2}v}{\partial y^{2}}+\frac{1-\mu }{2}\frac{\partial^{2}v}{\partial x^{2}}+\frac{1+\mu }{2}\frac{\partial^{2}u}{\partial x\partial y}-\mu \frac{\partial \varepsilon_{x}^{t}}{\partial y}-\frac{\partial \varepsilon_{y}^{t}}{\partial y})=0 \end{array}\right.$$

where *E* is the Young’s modulus, *μ* is the Poisson’s ratio, and *u* and *v* are the corresponding displacement components in the *x*- and *y*-directions. The *ε_x_^t^*, *α*(*T*) × *T*, denotes the thermal strain along the *x*-direction. The thermal expansion coefficient *α*, −4 × 10^−12^*T*^2^ + 8 × 10^−9^*T* + 4 × 10^−7^, varies with the temperature. The natural boundary condition is

(22) $$\left\{\begin{array}{c} \frac{E}{1-\mu^{2}}(n_{x}\frac{\partial u}{\partial x}+\mu n_{x}\frac{\partial v}{\partial y}+\frac{1-\mu }{2}n_{y}(\frac{\partial u}{\partial y}+\frac{\partial v}{\partial x})+n_{x}(\varepsilon_{x}^{t}+\mu \varepsilon_{y}^{t}))=0\\ \frac{E}{1-\mu^{2}}(n_{y}\frac{\partial v}{\partial y}+\mu n_{y}\frac{\partial u}{\partial x}+\frac{1-\mu }{2}n_{x}(\frac{\partial u}{\partial y}+\frac{\partial v}{\partial x})+n_{y}(\mu \varepsilon_{x}^{t}+\varepsilon_{y}^{t}))=0 \end{array}\right.$$

where the components of the normal vector are defined as *n_x_* and *n_y_* in the *x-* and *y*-directions. Additionally, essential boundary conditions are applied at the two ends of the actuator. For Equation (10), the discrete point **x***_I_* (*x_I_*, *y_I_*) determines the elements in the matrix **K***^I^*and vector *p^I^*.

Case I: If the point **x***_I_* is in the internal computational domain, **K***^I^* and *p^I^* should be derived based on Equation (21). The matrix **K***^I^* has the dimension of 2 × 2*N^l^*.

(23) $$\left\{\begin{array}{c} K_{1,2*j-1}^{I}=\frac{E}{1-\mu^{2}}(N_{I,xx}+\frac{1-\mu }{2}N_{I,yy})\\ K_{1,2*j}^{I}=\frac{E}{2(1-\mu )}N_{I,xy} \end{array}\right.$$

(24) $$\left\{\begin{array}{c} K_{2,2*j-1}^{I}=\frac{E}{2(1-\mu )}N_{I,xy}\\ K_{2,2*j}^{I}=\frac{E}{1-\mu^{2}}(N_{I,yy}+\frac{1-\mu }{2}N_{I,xx})\; \end{array}\right.$$

where **N***_I,xx_*, **N***_I,xy,_* and **N***_I, yy_* can also be obtained from the $\Phi_{I,xx}$, $\Phi_{I,xy}$, and $\Phi_{I,xy}$ based on Equation (8). For example, the $\Phi_{I,xy}$ can be expressed as

(25) $$\Phi_{I,xy}=\left[\begin{array}{llllllll} \frac{d^{2}R_{1}(x_{I})}{dxdy} & \frac{d^{2}R_{2}(x_{I})}{dxdy} & \cdots & \frac{d^{2}R_{N^{l}}(x_{I})}{dxdy} & 0 & 0 & 0 & 1 \end{array}\right]G^{-1}$$

and

(26) $$\left\{\begin{array}{c} p_{1}^{I}=\frac{E}{1-\mu^{2}}(\varepsilon_{Ix,x}^{t}+\mu \varepsilon_{Iy,x}^{t})\\ p_{2}^{I}=\frac{E}{1-\mu^{2}}(\mu \varepsilon_{Ix,y}^{t}+\varepsilon_{Iy,y}^{t}) \end{array}\right.$$

where $\varepsilon_{Ix,x}^{t}$ and $\varepsilon_{Iy,x}^{t}$ are the first derivative, with respect to *x,* of the thermal strain component (in the *x*- and *y*-directions) at the specific point **x***_I_*. Based on the temperature distribution, their value can be calculated by the central finite difference method. $\varepsilon_{Ix,y}^{t}$ and $\varepsilon_{Iy,y}^{t}$ both can be regarded as 0 in the light of the 1D model established in the electrothermal analysis.

Case П: If the point **x***_I_* belongs to the natural boundary, **K***^I^* and *p^I^* should be derived based on Equation (22).

(27) $$\left\{\begin{array}{c} K_{1,2*j-1}^{I}=\frac{E}{1-\mu^{2}}(n_{I_{x}}N_{I,x}+\frac{1-\mu }{2}n_{I_{y}}N_{I,y})\\ K_{1,2*j}^{I}=\frac{E}{1-\mu^{2}}(\mu n_{I_{x}}N_{I,y}+\frac{1-\mu }{2}n_{I_{y}}N_{I,x}) \end{array}\right.$$

(28) $$\left\{\begin{array}{c} K_{2,2*j-1}^{I}=\frac{E}{1-\mu^{2}}(\mu n_{I_{y}}N_{I,x}+\frac{1-\mu }{2}n_{I_{x}}N_{I,y})\;\\ K_{2,2*j}^{I}=\frac{E}{1-\mu^{2}}(n_{I_{y}}N_{I,y}+\frac{1-\mu }{2}n_{I_{x}}N_{I,x}) \end{array}\right.$$

where the formula of $\Phi_{I,x}$, adopted to reconstruct **N***_I,x_*, is

(29) $$\Phi_{I,x}=\left[\begin{array}{llllllll} \frac{dR_{1}(x_{I})}{dx} & \frac{dR_{2}(x_{I})}{dx} & \cdots & \frac{dR_{N^{l}}(x_{I})}{dx} & 0 & 1 & 0 & y_{I} \end{array}\right]G^{-1}$$

Other first-order derivatives of the shape function vector can be obtained by the same method, according to Equation (8).

(30) $$\left\{\begin{array}{c} p_{1}^{I}=\frac{E}{1-\mu^{2}}n_{Ix}(\varepsilon_{Ix}^{t}+\mu \varepsilon_{Iy}^{t})\\ p_{2}^{I}=\frac{E}{1-\mu^{2}}n_{Iy}(\mu \varepsilon_{Ix}^{t}+\varepsilon_{Iy}^{t}) \end{array}\right.$$

where *n_Ix_* and *n_Iy_* are components of the normal vector in the *x-* and *y*-directions at point **x***_I_*.

Case Ш: Otherwise, point **x***_I_* belongs to the Dirichlet boundary. To meet the coercive boundary condition, matrix **K***^I^* and *p^I^* are directly rewritten as

(31) $$K_{1,1}^{I}=K_{2,2}^{I}=1$$

and

(32) $$p_{1}^{I}=p_{2}^{I}=0$$

where the remaining entries in the local matrix **K***^I^* are zero. Finally, Equations (23), (24), (26)–(28), and (30)–(32) must be assembled into a global system of equations, analogous to Equation (12). In this global system, the stiffness matrix **K** and the force vector *P* have dimensions of 2*N^l^* × 2*N^l^* and 2*N^l^* × 1, respectively. For the element $K_{1,2*j-1}^{I}$, *I* represents the global index and *j* represents the local index. The corresponding global index *J* is defined for the point **x**_j_ in the whole problem domain. Accordingly, the element $K_{1,2*j-1}^{I}$ is placed in the (2*I* − 1)^th^ row and (2*J* − 1)^th^ column of the global stiffness matrix **K**. Similarly, the element $K_{2,2*j-1}^{I}$ belongs in the (2*I*)^th^ row and (2*J* − 1)^th^ column. The $p_{1}^{I}$ and $p_{2}^{I}$ are assembled into the (2*I* − 1)^th^ and (2*I*)^th^ row of the **P**. For the purposes of this analysis, the resulting global matrix equation can be solved efficiently using the direct iteration method.

## 5. Numerical Results and Discussions

A. Thermal measurement

**Figure 3:**
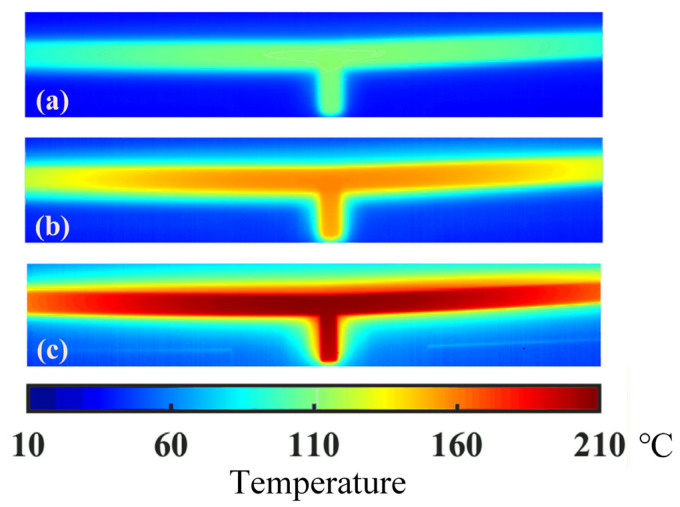
Experimental temperature distribution around the middle of the actuator under different voltages (**a**) −4 V, (**b**) −5 V, (**c**) −6 V.

For precise temperature acquisition, an infrared thermal microscope system with high spatial resolution was utilized. The system records thermal infrared images automatically at a frame rate of 200 Hz, each with a resolution of 392 × 258 pixels. A spatial calibration, performed with reference to the known dimensions of the ETA in the field of view, indicates that one pixel represents a physical distance of approximately 3.75 μm. As in the previous study, the high temperature existing in the middle area has a decisive impact on the performance of the actuator. Hence, the thermal image around the middle area should be preferentially captured in the experiment. Temperature distributions around the middle area under different voltages are measured and shown in Figure 3. As shown in the figure, the temperature decreases from the middle to either side of the actuator. When a voltage of 6 V is applied, the maximum temperature of about 210 °C lies in the central area. The thermal radiation may happen because the temperature near the actuator is higher than the room temperature.

B. Thermal prediction

**Figure 4:**
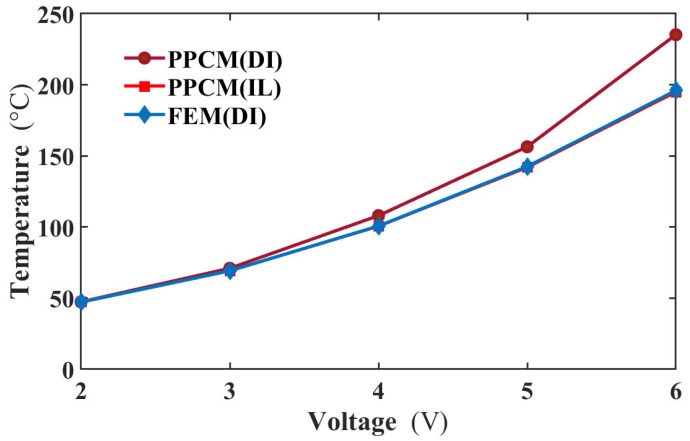
The middle temperatures of the actuator obtained by these three methods and FEM at different voltages.

To evaluate the performance of the incremental load (IL) method within the PPCM framework, its results were compared with those from the direct iteration (DI) method for solving Equation (10). Both sets of results were benchmarked against reference solutions obtained from the finite element method (FEM). Figure 4 presents the comparative results. As shown, the temperature difference calculated by the DI method increases significantly with applied voltage, reaching approximately 40 °C at 6 V. In contrast, the IL method demonstrates superior convergence within the PPCM. The temperature difference between the PPCM (IL) and FEM solutions remains within 1 °C across all voltage levels. Therefore, the integration of the IL method into the PPCM yields more accurate results in this study. This superiority can be attributed to the IL method’s enhanced capability in handling the material nonlinearity, where the thermal conductivity decreases as the temperature rises, while the resistivity has an opposite relationship with the temperature. These factors generate high nonlinearity in the equation. In addition, another interesting phenomenon is that the temperature result from FEM with DI is valid. That is to say, the FEM can be more stable than PPCM in numerical calculations.

**Figure 5:**
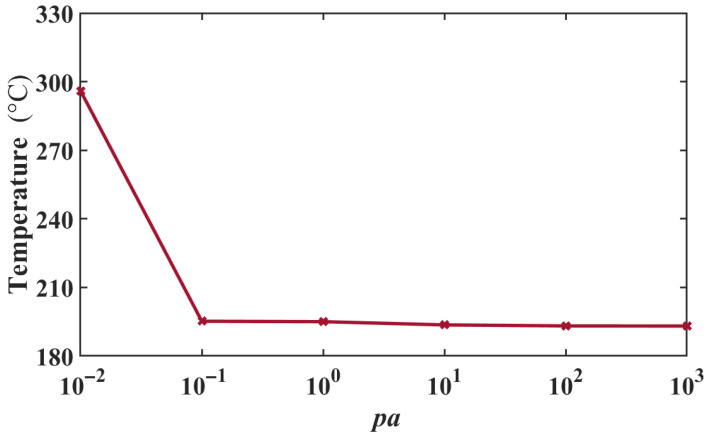
Maximum temperatures under 6 V varying with *p_a_* in MQ RBF.

**Figure 6:**
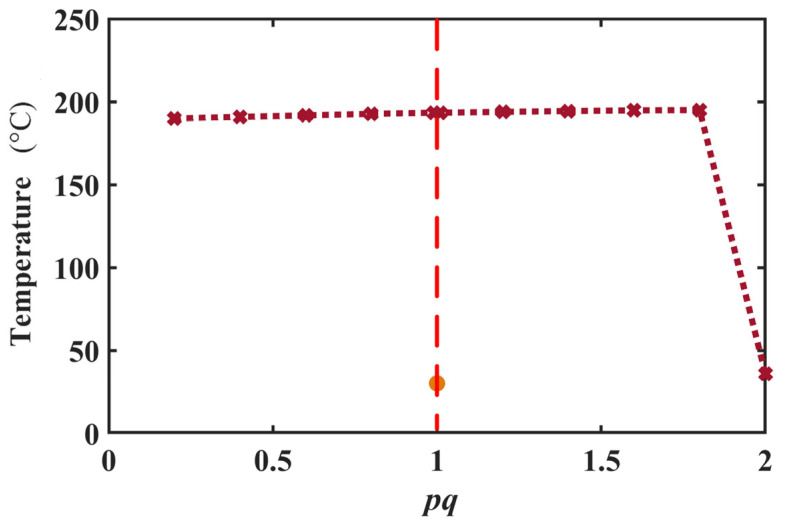
Maximum temperatures under 6 V vary with *p_q_* in MQ RBF.

To further explore the calculation stability of the PPCM (IL), the convergence test was performed. Three other types of RBF were employed in the PPCM to solve the equation, and the maximum temperature at 6 V is presented in Table 1. From the table, these three kinds of RBF have such poor performance that they may be unable to converge or solve the stagnation at the initial condition in the PPCM. The MQ RBF is best for this study. Based on the expression of the MQ RBF, its convergence analysis on two parameters, *p_a_* and *p_q_* was further conducted. The results are shown in Figure 5 and Figure 6, respectively. When pa varies over a wider scale range (10^−1^–10^3^), a better computational stability can be seen from Figure 5. However, the calculation diverges rapidly with *p_a_* close to zero. For *p_q_* in Figure 6, the parameter has a narrow range of computational convergence. Its calculation diverges rapidly when *p_a_* is close to 2. Moreover, a numerical singularity exists at the point *p_q_* = 1, in which MQ degenerates into a simple polynomial. In this case, its performance is similar to that of the cubic RBF. Thus, the value of *p_q_* should be far from 1. Finally, *p_a_* and *p_q_* are 1 and 1.8 for the electrothermal analysis.

**Figure 7:**
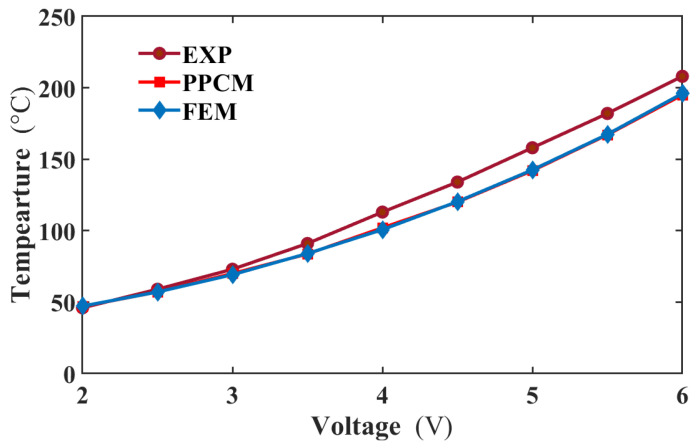
Middle temperatures comparison between the experiment, PPCM, and FEM from 2 to 6 V.

**Figure 8:**
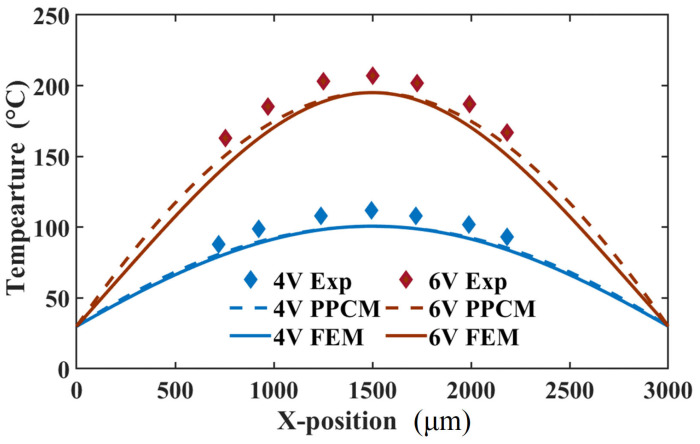
Comparison of the temperature distribution between the experiment, GFDM, and FEM at 4 and 6 V.

When the actuator is loaded with different voltages, its temperatures separately obtained from PPCM and FEM were compared with those from the experiment in Figure 7. The temperatures are extracted from the middle area of the actuator at the steady state. In this figure, the predicted temperature curve is in strong agreement with the FEM one. Furthermore, the PPCM results show reasonable agreement with experimental data, with a maximum temperature error of approximately 13 °C. For a more detailed evaluation, temperature profiles along the actuator length were extracted from thermal images. As shown in Figure 8, the temperature profiles predicted by PPCM agree well with FEM results at both 4 V and 6 V. The maximum discrepancy between PPCM and FEM is about 7 °C, located near the three-quarter point of the actuator at 6 V. However, compared to the experimental profile, the PPCM prediction shows a slight deviation. Specifically, a temperature difference emerges midway along the actuator and gradually increases towards both ends. As estimated from the curve at 6 V, the discrepancy between the PPCM and experimental temperature values is approximately 20 °C, corresponding to an approximate error of 10%. This can mainly be ascribed to the uncertain material parameters and excessive simplification of the boundary condition, where the Dirichlet boundary condition is employed.

C. Mechanical prediction

**Figure 9:**
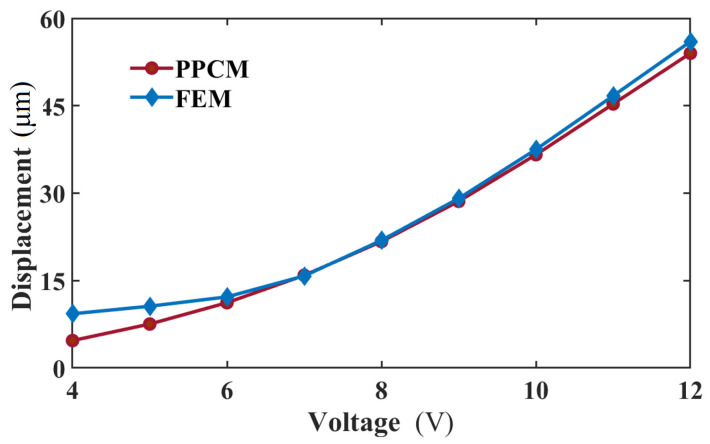
Maximum displacement of the actuator by the PPCM and FEM loaded with different voltages.

**Figure 10:**
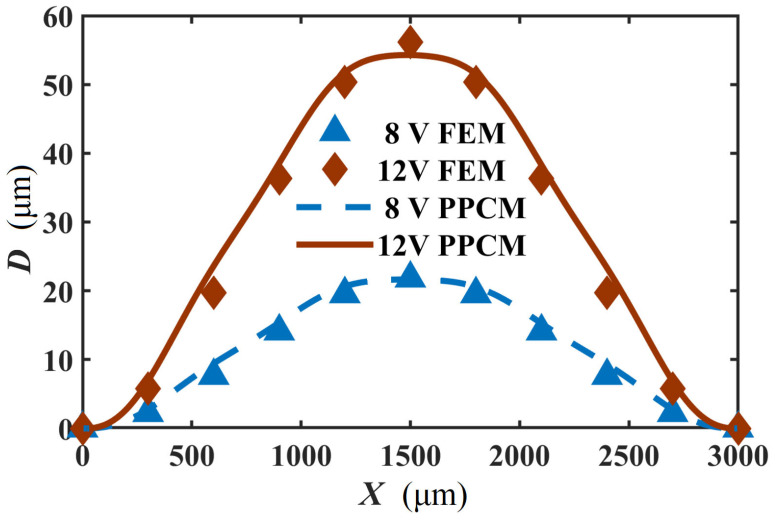
Comparison of displacement distribution along the length at 8 V and 10 V.

Figure 1c shows the node distribution of the 2D computational domain of the actuator. Here, the calculation stability of the PPCM for the thermomechanical analysis is explored. It was found that *p_a_* and *p_q_* are 1 and 1.03. Moreover, the radius of the support domain *d* = 46 μm is employed in the calculation. Under different voltages, the displacements in the middle area of the actuator, where the maximum displacement occurs, are calculated by the PPCM and FEM, are presented in Figure 9. In the figure, though a striking difference can be observed at lower voltages, the displacement curve of the PPCM is overall in good agreement with that of the FEM. The error of the displacement is merely about 2 μm when 12 V is applied. In our previous study [24], strong agreement was demonstrated between the same FEM model and experimental displacement data. When the V-shaped actuator is loaded with 8 V and 12 V, the displacement distribution along the length is presented in Figure 10. Based on the PPCM, the deflection at the sample points is in good agreement with that in the FEM curves. Given its good performance in the mechanical prediction, the PPCM can be employed to solve the thermomechanical model of the actuator.

## 6. Conclusions

This study presents a successful application of the Polynomial Point Interpolation Collocation Method (PPCM) to the coupled electrical, thermal, and mechanical analysis of a V-shaped microactuator. Using the PPCM, the thermal and mechanical behaviors of the actuator were evaluated independently. The temperature distribution obtained via PPCM shows good agreement with both finite element method (FEM) results and experimental data. Through computational stability analysis, the incremental load method is identified as particularly suitable for handling the strong nonlinearities inherent in the electrothermal model. Further comparison reveals, however, that the boundary conditions used in this study require refinement, as indicated by increasing temperature deviations from the middle toward both ends of the actuator. In terms of mechanical response, the displacement field computed with PPCM also aligns well with FEM predictions. These findings collectively demonstrate that the PPCM-based scheme provides a convenient, intuitive, and efficient implementation while avoiding high computational costs associated with the weak form of the meshless method. It is also validated that this scheme is more stable and accurate than other strong forms, such as GFDM and PINN, when dealing with multi-physics problems. The further acquisition and comparison of experimental displacement field data for this microactuator will be performed in the next step of our research.

In the context of topology optimization, PPCM completely avoids the computational instabilities and accuracy degradation associated with mesh distortion or remeshing in conventional finite element methods and strong-form meshfree approaches. PPCM also enables efficient and precise multi-physics coupling optimization by solving multiple governing equations in a unified manner within a single node-based framework, using distinct local approximating polynomials. As a node-based discretization method, PPCM generates structured data such as nodal field variables that are highly compatible with machine learning models, thereby facilitating the development of a hybrid intelligent optimization paradigm. In summary, PPCM provides a novel approach to topology optimization that overcomes mesh-related limitations and exhibits significant potential for applications in complex multi-physics systems and data-driven integration.

## Figures and Tables

**Table 1:** Temperature obtained from different kinds of RBF in the PPCM.

Name	Expression	Temperature/°C
MQ	${\left(1+p_{a}r^{2}\right)}^{p_{q}}$	195
Cubic	$1-6r^{2}+8r^{3}-3r^{4}$	30.15
Spline	$\exp(-cr^{2})$	30.18
CSRBF (Wl-C4)	${\left(1-r\right)}^{6}(3+18r+35r^{2})$	30.09

## Data Availability

All data are presented within the paper.
